# The Impact of Language Opacity and Proficiency on Reading Strategies in Bilinguals: An Eye Movement Study

**DOI:** 10.3389/fpsyg.2016.00649

**Published:** 2016-05-06

**Authors:** Diego de León Rodríguez, Karin A. Buetler, Noëmi Eggenberger, Marina Laganaro, Thomas Nyffeler, Jean-Marie Annoni, René M. Müri

**Affiliations:** ^1^Neurology Unit, Laboratory for Cognitive and Neurological Sciences, Department of Medicine, Faculty of Science, University of FribourgFribourg, Switzerland; ^2^Perception and Eye Movement Laboratory, Department of Neurology and Clinical Research, University of BernBern, Switzerland; ^3^Neuropsycholinguistic Team, Faculty of Psychology and Educational Sciences, University of GenevaGeneva, Switzerland

**Keywords:** language opacity, low proficiency, reading strategies, eye movements, bilingualism, reading aloud

## Abstract

Reading strategies vary across languages according to orthographic depth – the complexity of the grapheme in relation to phoneme conversion rules – notably at the level of eye movement patterns. We recently demonstrated that a group of early bilinguals, who learned both languages equally under the age of seven, presented a first fixation location (FFL) closer to the beginning of words when reading in German as compared with French. Since German is known to be orthographically more transparent than French, this suggested that different strategies were being engaged depending on the orthographic depth of the used language. Opaque languages induce a global reading strategy, and transparent languages force a local/serial strategy. Thus, pseudo-words were processed using a local strategy in both languages, suggesting that the link between word forms and their lexical representation may also play a role in selecting a specific strategy. In order to test whether corresponding effects appear in late bilinguals with low proficiency in their second language (L2), we present a new study in which we recorded eye movements while two groups of late German–French and French–German bilinguals read aloud isolated French and German words and pseudo-words. Since, a transparent reading strategy is local and serial, with a high number of fixations per stimuli, and the level of the bilingual participants’ L2 is low, the impact of language opacity should be observed in L1. We therefore predicted a global reading strategy if the bilinguals’ L1 was French (FFL close to the middle of the stimuli with fewer fixations per stimuli) and a local and serial reading strategy if it was German. Thus, the L2 of each group, as well as pseudo-words, should also require a local and serial reading strategy. Our results confirmed these hypotheses, suggesting that global word processing is only achieved by bilinguals with an opaque L1 when reading in an opaque language; the low level in the L2 gives way to a local and serial reading strategy. These findings stress the fact that reading behavior is influenced not only by the linguistic mode but also by top–down factors, such as readers’ proficiency.

## Introduction

Reading strategies depend on the language being read (Frost, 2012). Since the rules for converting the written code (graphemes) into oral production (phonemes, i.e., the smallest meaningful units of written language into their analogies in spoken language) vary across languages, different languages should also engage distinct behavioral and neural reading processes. Based on this assumption the “Orthographic Depth Hypothesis” (Katz and Feldman, 1983; Katz and Frost, 1992) postulates that different strategies are involved in reading languages with varying degrees of opacity. Transparent languages such as Spanish and German, in which the majority of the words follow simple grapheme–phoneme conversion rules (GPC), should be read differently from opaque languages such as English and French, in which most of the words follow ambiguous GPC (Ziegler et al., 2001; Seymour et al., 2003; Simon et al., 2006; Bar-Kochva and Breznitz, 2012; Joshi et al., 2012).

In line with this hypothesis, the Dual Route Cascaded Model states that, after pre-lexical unit identification, word pronunciation is based on two routes of processing (Coltheart et al., 2001; Taylor et al., 2013). Reading aloud is achieved via a lexical route for words with complex GPC, in which the rules for generating phonemes from graphemes are likely to be established by lexical-semantic knowledge. In contrast, reading words with simple GPC and non-words would rely on a non-lexical route to convert graphemes into their corresponding phonemes. The model also states that each route implies a specific reading strategy to give way to the phonology. Therefore, using the lexical route implies global and parallel word processing, while using the non-lexical route implies that words and non-words are processed locally and serially from left to right. Thus, the model envisages a cascaded principle corresponding to an activation of both routes; consequently phonology is always partially assembled and partially lexical, but not necessarily fully specified by both routes.

The different reading strategies for opaque versus transparent languages can be advantageously assessed by focusing on eye movement patterns (Rayner, 2009). However, only a few studies have directly assessed eye movements across different languages (Fukuda and Fukuda, 2009). In light of the research across languages with different orthographic depths, it is reasonable to expect that if in transparent languages the reading strategy is local and serial, and in opaque languages it is global and parallel, then this should have an impact on the oculomotor patterns when reading. Since these theoretical assumptions suggest variations between the two strategies in terms of lexical and phonological requirements, and the oculomotor measures in reading have also proved to be modulated by the same factors, then the lack of data in this field is surprising. Bar-Kochva and Breznitz (2012) pointed out that one of the major reasons for this lack of data is the high level of difficulty in controlling inter-subject confounding factors, which may compromise the comparison between opaque and transparent languages.

Assuming a close relationship between language orthographic depth and oculomotor patterns in reading, modulations in the first fixation location (FFL) in word reading should be expected in readers from different orthographic depth languages. Recently, Rau et al. (2015) captured the oculomotor behavior of German and English children and adult speakers (all monolinguals) while they were reading aloud sentences in their corresponding transparent or opaque language. The equivalent cross-linguistic sentences contained a target stimulus varying in length (short and long) and lexical status (low frequency words, high frequency words, and pseudo-words). They found that for the same time spent in word processing, German children differed from English children in the way of solving the task, while in adults, English speakers processed pseudo-words for longer durations than their German counterparts. In addition, even though all adult readers spent more time processing long than short pseudo-words, this length effect was more pronounced in English than in German readers. The authors explained this issue in the sense that both groups were using the same reading strategy for processing pseudo-words, namely by processing small-units, but because German readers have more experience in the use of such a “local” reading strategy they were faster than the English readers. Indeed, for English readers the preferred reading strategy has been advanced to “globally” process large-units (see Ziegler and Goswami, 2005). Consequently, in opaque as compared with transparent languages, the reading strategy should be more “global,” and thus the FFL might be closer to the center of words, as variations in the FFL and its duration are modulated by the lexical frequency (Vitu et al., 1990; Yao-N’dre et al., 2013). On the other hand, infrequent words or words with orthographic ambiguities present an FFL closer to their beginning (Clark and O’Regan, 1999), and shorter saccade length and an FFL close to the beginning of words were associated with the engagement of a serial sub-lexical decoding at the single word reading (Hawelka et al., 2010). Both issues support the hypothesis of a “local” reading strategy that can also be associated with transparent languages. In sum, more local word processing in transparent as compared with opaque languages can thus be associated with a leftward-shifted FFL.

Bilinguals seem to be good models for evaluating the impact of language opacity on reading strategies. The first study modulating the effects of language opacity on reading strategies by means of eye movement patterns in bilinguals was recently conducted in our laboratory (de León Rodríguez et al., 2015). In this study, a group of early French–German bilinguals (bilinguals under the age of seven), who had the same reading level in both languages, were required to read aloud isolated words and pseudo-words (linguistically legal non-words). While they were reading, the FFL, its duration, and latency for sending the first saccade to the stimulus were measured. The results showed a local reading strategy associated with words (FFL close to the beginning) in the transparent language (German) and a global reading strategy (FFL less close to the beginning of words) in the opaque language (French). In addition, the FFL between words and pseudo-words only differed in the opaque language, which would indicate that the absence of lexical representation in linguistically legal stimuli (pseudo-words) was also processed using a local reading strategy. In line with recent data (Rau et al., 2015), this study supports the finding that the critical factor in explaining differences in reading behavior indexed by eye movement patterns across languages is their degree of opacity.

In addition to the orthographic depth factor, language proficiency has also been shown to influence whether global lexical or local non-lexical reading strategies are engaged for reading. Jeon (2012) demonstrated that when reading ambiguous words in sentences written in an opaque language, readers with a high proficiency level in that opaque language solicited more brain regions supporting the lexical route (the anterior cingulate cortex, middle frontal, and fusiform gyri). Readers with low proficiency in the same opaque language solicited preferential brain regions supporting the non-lexical route (the inferior parietal lobe and the inferior frontal gyrus). These results can be interpreted in terms of the link between word forms and their lexical representations being stronger in the high-proficiency readers than in the low-proficiency readers, which determines the reading strategy (lexical or non-lexical). In support of this assumption, reading less frequent words and pseudo-words has been associated with the use of the non-lexical route (Proverbio et al., 2004; Heim et al., 2005), whereas the engagement of the lexical route increases when reading highly familiar words (Fisher et al., 2012). Weaker lexical activation has also been associated with the second language (L2) compared with the first language (L1) of bilinguals in the literature on eye movement (Gollan et al., 2008; Gollan et al., 2011), which also suggests that the L2 might favor a local reading strategy.

Hence, two aspects seem to play a crucial role in whether global or local reading strategies are engaged: the opacity of the language and the proficiency level of the reader. In bilinguals, the choice of a particular reading strategy can thus be modulated by the opacity of the language and the proficiency level of the reader. Therefore, a bilingual with a transparent L1 and a low proficiency level in his/her L2 would preferentially use a non-lexical (local) reading strategy for both of his/her languages, while a bilingual with an opaque L1 and a low proficiency level in his/her L2 would use a lexical (global) reading strategy for his/her L1 but a non-lexical reading strategy for his/her L2. This hypothesis, however, lacks empirical support and is the focus of the present study.

To address this question, we asked two groups of French–German and German–French bilinguals (L1-L2 respectively) with a low proficiency level in their L2 to read aloud isolated words and pseudo-words in each of their languages. The landing position of the FFL, the first fixation duration (FFD), the latency for sending the saccade previous to the first fixation (LSS), and the number of fixations (or fixation count, FC) per stimulus were recorded by studying their eye movement behavior. The FC was compared to gaze duration (or GD, which consists of the sum of all fixation durations in a word), as both measures represent variations in eye fixation times in reading (Godfroid, 2012). However, the FC was chosen instead of GD based on the assumption that serial reading processing is associated with the use of the non-lexical route (Coltheart et al., 2001). In terms of the oculomotor measures, the FFD and LSS were not expected to vary, as task demands were the same (to read aloud). On the contrary, the FFL and FC were expected to be the most sensitive measures of the use of a local or global reading strategy. Thus, our first aim was to confirm our previous results, i.e., a local versus a global reading strategy (FFL close versus less close to the beginning of words), which was associated with transparent versus opaque languages, respectively. The second aim was to test the impact of a low proficiency level on the reading strategy selection process.

For the first aim, we hypothesized that since the bilinguals’ proficiency level is lower in their L2 than in their L1, the impact of language opacity should be observed in their L1. Correspondingly, the FFL should be closer to the beginning of the words, and the FC higher, when German-L1 bilinguals are reading in the transparent language (German) than when French-L1 bilinguals are reading words in the opaque language (French).

For the second aim, the prediction was that because the bilinguals’ proficiency level is lower in their L2 than in their L1, reading in the L2 should favor a local and serial method of word processing. Yet, as German-L1 bilinguals are already using a local and serial reading strategy, the difference between both bilingual groups may only be observed in the opaque language (French). Therefore, in French, the FC should be higher and the FFL closer to the beginning of the words for the bilinguals who are reading in their L2 compared with those reading in their L1. More importantly, no difference between groups is expected when the bilinguals are reading in German.

Finally, pseudo-words are related to the use of the non-lexical route (as for transparent languages); they should therefore be processed using a local and serial reading strategy. Since we postulated that French-L1 bilinguals would rely heavily on the lexical route in the opaque language (French), a longer FC for pseudo-words compared with words is expected only in this opaque language and only for this specific group of bilinguals.

## Materials and Methods

### Participants

Forty L1-dominant bilinguals (mean age = 24.11 years, *SD* = 3.51, 26 women) participated in the study. Half of them had French as their first language (L1) and German as their second (L2), and the rest had the reverse pattern. The inclusion criteria were having L1 as their dominant language and having a low proficiency level in their L2 (see below). All participants had normal or corrected-to-normal vision and were unaware of the research hypotheses. They were students from the Universities of Fribourg and Bern, Switzerland, and were paid to participate in the study. The local Ethics Committee approved all the research procedures.

The level of proficiency in both languages was evaluated using a lexical decision task from the DIALANG (Zhang and Thompson, 2004). Participants’ history of bilingualism as well as their current percentages of L1 and L2 exposure were measured by means of the Language Experience and Proficiency Questionnaire (LEAP-Q, Marian et al., 2007). The lexical decision task from the DIALANG is a preliminary-level test giving a score from 0 to 1000 (i.e., 0–100: knowledge of very few words; 101–200: very basic knowledge; 201–400: a limited vocabulary; 401–600: a good basic vocabulary; 601–900: an advanced level with a very substantial vocabulary; and 901–1000: a native speaker level). The LEAP-Q is a self-reporting questionnaire used for measuring bilingual language status. The questionnaire evaluates language competence (proficiency, dominance, and preference), age of language acquisition, means of language acquisition, and past and present language immersion. **Table 1** shows the bilingual profile of the participants.

**Table 1:** Bilingual language status assessed by DIALANG and LEAP-Q between languages (L1, L2).

	L1		L2		
Profile measures	Mean ± SD	Range	Mean ± SD	Range	*p*
DIALANG					
Level test^a^	896.60 ± 93.58	600–1000	226.53 ± 271.35	0–899	∗
LEAP-Q					
Current language exposure (%)	64.15 ± 20.71	25–100	18.63 ± 16.47	0–70	∗
Self-reported proficiency^b^					
Understanding	9.83 ± 0.50	8–10	6.70 ± 2.23	2–10	∗
Speaking	9.58 ± 0.75	7–10	5.83 ± 2.54	1–10	∗
Reading	9.65 ± 0.74	7–10	6.55 ± 1.97	2–10	∗
Age milestones (years)					
Started learning	0.30 ± 1.02	0–5	9.25 ± 2.42	0–14	∗
Attained fluency	2.70 ± 1.09	1–7	15.59 ± 3.93	5–25	∗
Started reading	5.75 ± 1.13	4–8	11.78 ± 2.85	8–19	∗
Attained reading fluency	7.51 ± 1.34	5–10	15.50 ± 2.93	10–23	∗
Immersion duration (years)					
Country/Swiss canton^c^	22.63 ± 3.69	11–33	7.99 ± 9.30	0–28	∗
Family	21.55 ± 5.94	0–33	2.34 ± 6.18	0–26	∗
School	18.51 ± 4.63	10–28	3.93 ± 4.23	0–12	∗
Contribution to language learning^d^					
From family	9.35 ± 2.01	1–10	2.43 ± 3.48	0–10	∗
From friends	8.70 ± 1.91	0–10	5.70 ± 3.97	0–10	∗
From reading	8.25 ± 1.86	3–10	5.23 ± 2.38	0–10	∗
From TV	5.70 ± 3.23	0–10	2.60 ± 2.43	0–8	∗
From radio	3.78 ± 2.94	0–10	2.20 ± 2.43	0–8	∗
From self -instruction	2.08 ± 2.96	0–10	2.70 ± 2.96	0–10	–
Extent of language exposure^e^					
To family	8.70 ± 2.73	0–10	1.05 ± 1.84	0–9	∗
To friends	8.90 ± 1.58	3–10	4.28 ± 3.48	0–10	∗
To reading	7.10 ± 1.71	4–10	3.98 ± 2.95	0–10	∗
To TV	6.73 ± 3.04	0–10	1.58 ± 1.57	0–6	∗
To radio	4.88 ± 2.85	0-10	2.15 ± 2.12	0–8	∗
Self -instruction	0.65 ± 1.82	0–10	1.03 ± 1.61	0–5	–
Self-reported foreign accent^f^					
Perceived by self	0.03 ± 0.16	0–1	5.13 ± 2.39	1–10	∗
Identified by others	0.03 ± 0.16	0–1	6.63 ± 3.43	1–10	∗

^∗^*p < 0.05.*^a^*Range: 0–100 (low vocabulary level) to 901–1000 (native speaker level);*^b^*range: 0 (none) to 10 (perfect);*^c^*It concerns a Swiss canton and/or a country where the tested language was spoken;*^d^*range: 0 (not a contributor) to 10 (most important contributor);*^e^*range: 0 (never) to 10 (always);*^f^*range: 0 (none) to 10 (pervasive).*

### Material

The stimuli consisted of 160 words (80 five-letter and 80 eight-letter nouns) per language and 60 pseudo-words (30 five-letter and 30 eight-letter pseudo-words). The pseudo-words were orthographically and phonologically legal across languages, so that the same pseudo-words were presented in French and German. The words were equivalent between the languages in terms of summated position-nonspecific bigram frequency, log-transformed lexical frequency and neighborhood size (the characteristics of the stimuli are presented in Supplementary Table S1). Furthermore, all of them were presented without diacritics, in uppercase, and using Courier New 72 pt. in bold as a font. For a full and detailed description of how the words were selected and how the pseudo-words were produced, please refer to de León Rodríguez et al. (2015).

### Apparatus

The experiment was designed, executed and analyzed using the SMI Experiment Suite^TM^ system (Sensomotoric Instruments GmbH, Teltow, Germany), and a video-based dark-pupil tracking system (SMI iView X^TM^ RED) was used for eye movement recording. The system had a temporal resolution of 250 Hz (sampling rate) and a spatial resolution of 0.03°, and it was able to compensate for head movements. A calibration procedure was performed using the 13-point calibration option. The procedure was run on a screen of 22” in size with a resolution of 1680 × 1050 pixels, 32-bit color depth, and a refresh rate of 60 Hz.

### Procedure

The experiment was conducted in a quiet room. In accordance with the system’s specifications, participants were placed 60–80 cm. in front of the experimental screen. Their heads were free, but any body movement was discouraged.

Participants performed both language tests in the same session, separated by a 15-min break. Both procedures were completely equivalent, differing only in the language in which they were written (French and German). The French and German procedures lasted approximately 25 min each. These were characterized by a Reading Aloud Activation part, an Instruction and Training part, and a Testing part. The order of testing for each language (language order) was counterbalanced, taking the participant’s L1 into account. Eight calibrations were involved in each procedure (one at the beginning of each procedure, six in the Testing part, and one at the end).

Two well-trained experimenters tested each language using exactly the same procedure, one speaking fluent French and the other speaking fluent German. In order to increase the linguistic mode activation, the participants started each procedure by reading aloud a text with a high level of difficulty for 3 min (the Reading Aloud Activation part): “Boule de Suif” in French (Maupassant, 1900) and “Casanovas Heimfahrt” in German (Schnitzler, 1918). After the participant read aloud the text, the instructions were presented followed by the training parts.

In the Instructions and Training parts, participants were informed as to how to perform the task and were told that the words were written in uppercase, without diacritics. In order to allow participants to be focused on the Reading Aloud task (and not on the Lexical Decision task), we omitted to tell them about the existence and repetition of pseudo-words between languages. Instead, they were informed that some words were extremely common and others extremely rare. Whenever necessary, additional information was given in the language of evaluation. Before starting the task, participants were given five practice words, which differed from the experimental stimuli.

The Testing part consisted of six blocks, in which the stimulus categories were organized in a pseudo-randomized order at a rate of one pseudo-word per two or three words. There were six blocks in total with 10 pseudo-words each, four of them with 27 and two of them with 26 words. All of the stimuli were presented randomly while respecting these categories. The structure of each block comprised a rest period in which participants closed their eyes for 30s, followed by the performance of a calibration and then by the task. Only during the rest periods and calibration measures was the experimenter allowed to interact with participants.

**FIGURE 1:**
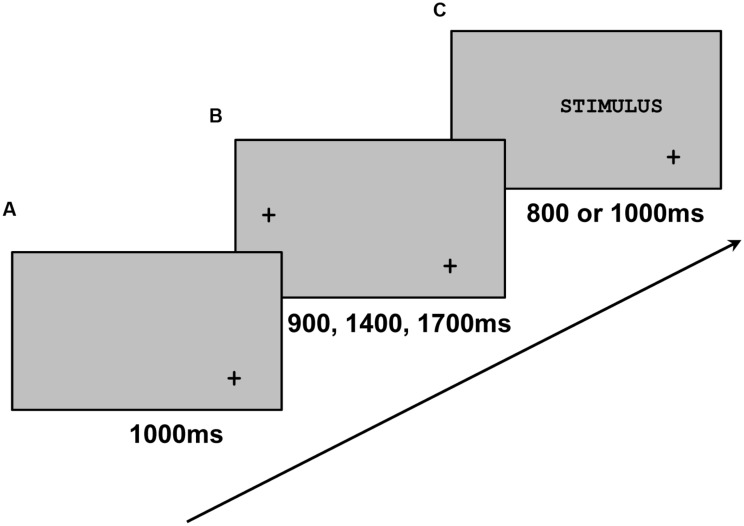
**Illustration of the way in which each stimulus was presented**. The down-cross was presented alone **(A)** for 1000 ms, with the left-cross **(B)** randomly at 900, 1400, or 1700 ms, and with the stimulus **(C)** for 800 ms for five-letter stimuli or 1000 ms for eight-letter stimuli. The timeline is represented by the arrow. Adapted from de León Rodríguez et al. (2015, p. 6, Figure 1).

**Figure 1** shows how each stimulus was presented per trial. For each trial, participants were instructed to fixate on the down-cross when it was alone (A in **Figure 1**), then the left-cross (B in **Figure 1**), and finally they had to read aloud the stimulus on their right (C in **Figure 1**) as naturally as possible and go back to the down-cross. The down-cross remained present throughout the trial, and the distance between the left-cross and the beginning of the stimuli was always a visual angle of 10.3°. The left-cross (B in **Figure 1**) appeared at different time intervals in order to avoid anticipation (i.e., saccades starting before the stimulus onset).

After being tested in both languages, participants were debriefed (they were informed about the existence of pseudo-words and their repetition between languages), and the lexical decision task from the DIALANG was performed (starting with the last language tested in the procedure).

### Dependent Variables

The main measure was the FFL within the stimulus (FFL, defined as the proportion between the stimulus length and the position of the first fixation along the stimulus, being 0, 50, and 100% at the beginning, the middle, and the end of the stimulus, respectively). A fixation was considered to be the first if its previous saccade started where the left-cross was situated (B in **Figure 1**). FFL outside the initial part of the stimulus and anticipatory eye movements were considered errors and excluded from the analyses (7.1%). From there, three additional oculomotor measures were calculated: the latency between the stimulus onset and the beginning of the first saccade (LSS, in milliseconds), the FFD (in milliseconds), and the total number of fixations per stimulus processed (FC).

### Design

To test our three hypotheses, a 2 by 2 by 2 design with language of reading (French vs. German), participants’ L1 (French-L1 vs. German-L1), and lexicality (words vs. pseudo-words) as factors was applied to each measure. In addition, to exclude any bias we included the current L2 exposure (as a percentage), the age of L2 acquisition, and the language testing order (German to French vs. French to German) as covariates. The statistical models do not include the stimulus length factor (five- and eight-letter stimuli). However, it is worth noting that the results are not explained by a specific stimulus length. Indeed, the analyses were applied to each length category separately and the main results are equivalent in each of them.

### Data Analyses

Statistical analyses were carried out using the R software (version 3.0.3; R Development Core Team, 2014). To address the principal aim of the present study, i.e., reading strategies across language opacity, the same linear mixed-effect model (LMEM) was applied to each oculomotor measure using the lme4 package (Baayen et al., 2008; Bates et al., 2014). The model was set in accordance with the theoretical research hypothesis, and the choice of applying the same LMEM to several measures was established in line with previous studies linking eye movement behavior to reading (Whitford and Titone, 2012, 2015; Whitford et al., 2013). Significant effects for all fixed factors were based on the convention of a *t*-value above 1.96 and *p*-value (calculated from *F*-test) below 0.05 based on Satterthwaite’s approximations for denominator degrees of freedom (implemented in the lmerTest package, version 2.0-6).

The fixed part of the model comprised participants’ L1 (French-L1 vs. German-L1), language of reading (French vs. German), lexicality (words vs. pseudo-words) and, as control predictors, language testing order (German to French vs. French to German), current L2 exposure (as a percentage), and age of L2 acquisition. The random part of the model included items and participants with random intercepts and random slope adjustments for the language of reading (Baayen, 2008; Barr et al., 2013).

## Results

### Distribution of the First Fixation Location

**FIGURE 2:**
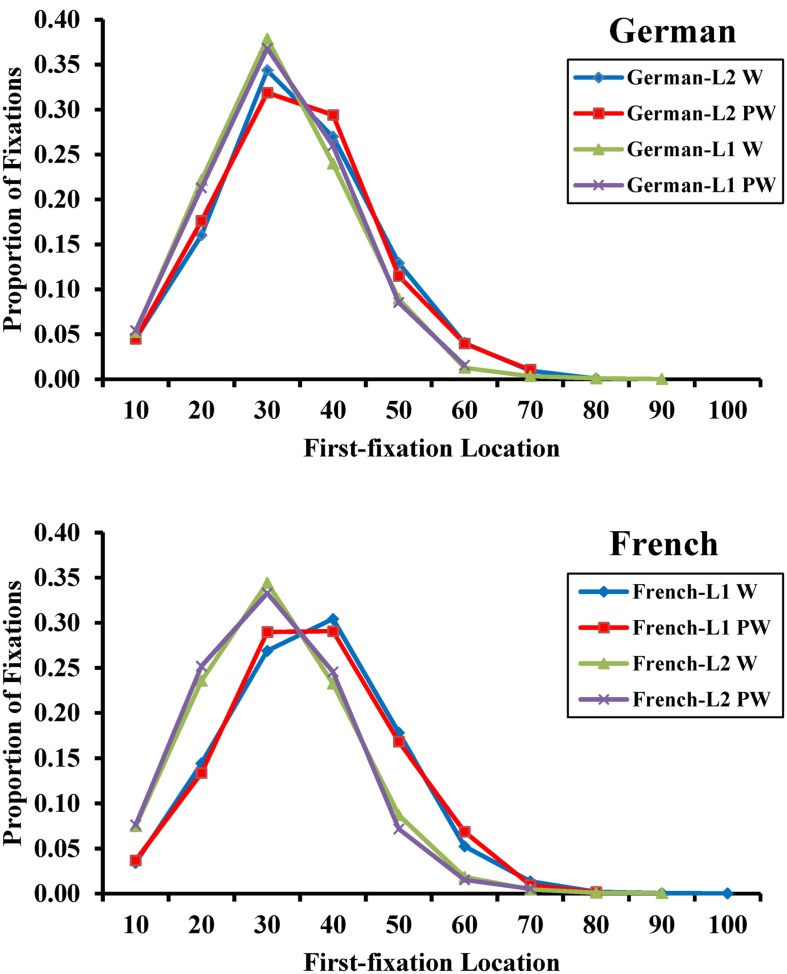
**The FFL curves in German (up graph) and French (bottom graph) reading**. Words are presented in blue lines for French (L1) and German (L2) bilinguals and in green for German (L1) and French (L2) bilinguals. Pseudo-words are presented in red lines for French (L1) and German (L2) bilinguals and in violet for German (L1) and French (L2) bilinguals.

Before presenting the oculomotor effects, **Figure 2** presents the distribution of the FFL for words and pseudo-words across groups. The figure shows the FFL data in each language mode separately. The FFL results of French and German were in agreement with previous results in single-word reading tasks (Vitu et al., 2004).

### Reading Strategies in Bilinguals’ Transparent (German) and Opaque (French) Languages

For ease of comprehension, the means of the participants’ oculomotor measures are presented in **Table 2** and next to the output in the text.

**Table 2:** Mean values per words, pseudo-words, language of reading (German, French), and first and second languages (L1, L2) of participants for FFL (%), FC (#), LSS (ms), and FFD (ms).

		German		French	
		L1	L2	L1	L2
FFL	Words	27.02	29.85	32.34	26.50
		[26.63;27.41]	[29.42;30.27]	[31.90;32.78]	[26.08;26.91]
	Pseudo-Words	27.34	29.69	32.12	25.92
		[26.70;27.97]	[28.99;30.39]	[31.40;32.85]	[25.26;26.59]
FC	Words	2.77	2.66	2.48	2.84
		[2.74;2.80]	[2.63;2.69]	[2.45;2.51]	[2.81;2.87]
	Pseudo-Words	2.85	2.74	2.65	2.88
		[2.80;2.89]	[2.69;2.78]	[2.61;2.70]	[2.83;2.93]
LSS	Words	161.40	169.20	169.30	161.10
		[159.27;163.55]	[167.11;171.21]	[167.37;171.24]	[159.25;162.98]
	Pseudo-Words	161.30	170.20	170.10	162.00
		[157.86;164.65]	[166.86;173.64]	[166.95;173.16]	[158.92;165.17]
FFD	Words	193.60	211.20	202.20	197.40
		[190.85;196.43]	[208.10;214.24]	[199.24;205.11]	[194.63;200.23]
	Pseudo-Words	195.60	205.90	208.20	199.90
		[190.84;200.44]	[200.86;210.92]	[203.07;213.31]	[195.25;204.56]

*Values in brackets are 95% confidence intervals. FFL, first fixation location; FC, fixation count; LSS, latency for sending the saccade before FFL; FFD, first fixation duration.*

The LMEM analyses showed several significant two-way and three-way interactions. In addition, there was a significant main effect of participants’ L1 on FFL [*F*(1,38.8) = 3.98, *p* = 0.053] and FC [*F*(1,40.2) = 5.66, *p* = 0.022], as FFL was closer to the beginning of the stimuli (26.72 vs. 31.05%) with higher FC for bilinguals with German-L1 as compared with bilinguals with French-L1 (2.82 vs. 2.60). From the control predictors, there was a main effect of current L2 exposure for FFD [*F*(1,40) = 6.29, *p* = 0.016], participants, who were less exposed to their L2, produced longer FFD. **Table 3** presents *β* and *t*-values for the same analyses for LSS, FFL, FFD, and FC. According to the prediction made at the end of the introduction section, we focused first on FFL and FC.

**Table 3:** βs, standard errors (SEs), and *t*-values for LMEM of the latency for sending the first fixation (LSS, millisecond), the first fixation location (FFL, %), the first fixation duration (FFD, millisecond), and the fixation count (FC) of stimuli.

	LSS			FFL			FFD			FC		
	β	*SE*	*t*	*β*	*SE*	*t*	β	*SE*	*t*	β	*SE*	*t*
Fixed effects												
L1^1^	−7.00	9.05	−0.77	**−5.82**	**2.35**	**−2.48^∗^**	−2.10	9.75	−0.22	**0.36**	**0.09**	**4.16^∗^**
LRead^2^	−0.57	3.32	−0.17	**−2.57**	**0.86**	**−2.97^∗^**	8.67	4.63	1.87	**0.19**	**0.06**	**3.25^∗^**
Lexicality^3^	1.33	1.60	0.83	−0.26	0.53	−0.48	5.98	3.09	1.94	**0.17**	**0.07**	**2.49^∗^**
L1 X LRead	0.60	4.70	0.13	**3.06**	**1.14**	**2.68^∗^**	−12.15	6.30	−1.93	**−0.25**	**0.04**	**−5.76^∗^**
L1 X Lexicality	−0.88	2.29	−0.38	−0.33	0.45	−0.74	−3.63	3.70	−0.98	**−0.13**	**0.03**	**−4.35^∗^**
LRead X Lexicality	−0.18	2.27	−0.08	0.03	0.55	0.05	**−11.26**	**3.96**	**−2.84^∗^**	−0.09	0.06	−1.65
L1 X LRead X Lexicality	−0.41	3.25	−0.13	0.94	0.63	1.48	**10.76**	**5.25**	**2.05^∗^**	**0.12**	**0.04**	**2.82^∗^**
Control predictors
Age of L2 acquisition^4^	−1.07	1.90	−0.56	0.38	0.43	0.90	1.45	1.79	0.81	−0.02	0.02	−1.43
Current L2 exposure^5^	−0.46	0.28	−1.65	0.00	0.06	−0.02	**−0.67**	**0.27**	**−2.51^∗^**	0.00	0.00	0.41
Testing order^6^	−2.08	8.96	−0.23	−2.67	2.01	−1.33	−12.57	8.46	−1.49	0.13	0.08	1.61
Intercept	22.35	20.26	1.10	1.23	4.62	0.27	4.69	19.44	0.24	−0.09	0.19	−0.47

	**LSS variance**			**FFL variance**			**FFD variance**			**FC variance**		
	**Intercept**		**Slope (LRead)**	**Intercept**		**Slope (LRead)**	**Intercept**		**Slope (LRead)**	**Intercept**		**Slope (LRead)**

Random effects												
Participants	786.50		191.70	53.59		11.92	895.94		321.73	0.07		0.01
Items	0.00			7.95		0.24	121.51		32.86	0.18		0.00
Residual		2131.00			81.34			5571.87			0.35	

*L1, first-language; L2, second-language; LRead, language of reading; LMEM, linear mixed effect models.*^1^*Contrasts were treatment coded (German-L1 vs. French-L1); model assumes French-L1 as the baseline.*^2^*Contrasts were treatment coded (German vs. French); model assumes French as the baseline.*^3^*Contrasts were treatment coded (Words vs. Pseudo-Words); model assumes Words as the baseline.*^4^*Continuous (years).*^5^*Continuous (% time).*^6^*Contrasts were treatment coded (German to French vs. French to German order); model assumes French to German order as the baseline.*^∗^*and in bold p < 0.05.*

Moreover, as can be seen in **Table 3**, there are several significant two-way and three-way interactions for FFL, FFD, and FC [for FFL a participants’ L1 by language of reading interaction, *F*(1,41.2) = 9.63, *p* = 0.003; for FFD a language of reading by lexicality interaction, *F*(1,162.1) = 3.81, *p* = 0.053 and a triple interaction, *F*(1,15867.9) = 4.21, *p* = 0.040; for FC a participants’ L1 by language of reading interaction, *F*(1,44.3) = 19.91, *p* < 0.001, a participants’ L1 by lexicality interaction, *F*(1,15842.2) = 11.01, *p* < 0.001, and a triple interaction, *F*(1,15851.5) = 7.94, *p* = 0.005]. To decompose the two-way and three-way interactions and therefore simplify their interpretation, sub-analyses in French and German were calculated separately. In addition, this choice was also motivated by both the number of interactions in which the language of the reading factor was implicated and its coherence with our hypothesis, i.e., testing the impact of language opacity on reading strategies between bilinguals. Finally, in order to simplify the presentation of the results and render them more coherent, the participants’ L1 will be expressed in terms of French-L1 vs. French-L2 and German-L1 vs. German-L2 for each language, respectively, to differentiate between the groups.

### Reading Strategies in the Transparent Language (German) of Bilinguals

**Table 4** presents the LMEM output in German of LSS, FFL, FFD, and FC. The control predictor of current L2 exposure was observed to have a significant effect only for FFD [*F*(1,39.9) = 5.47, *p* = 0.024], indicating that participants with less current exposure to their L2 presented longer FFD. As expected, no other significant effect or interaction was present in this language of reading. It is therefore in the next sub-analyses (in French) where it is expected to have effects that end the description of the two-way and three-way interactions evoked above in the principal LMEM analyses.

**Table 4:** German – βs, standard errors (SEs), and *t*-values for LMEM of the latency for sending the first fixation (LSS, millisecond), the first fixation location (FFL, %), the first fixation duration (FFD, millisecond), and the fixation count (FC) of stimuli.

	LSS			FFL			FFD			FC		
	β	*SE*	*T*	β	*SE*	*t*	β	*SE*	*t*	β	*SE*	*t*
Fixed effects												
L1^1^	−6.67	10.21	−0.65	−2.78	2.03	−1.37	−14.45	8.70	−1.66	0.11	0.08	1.27
Lexicality^2^	1.16	1.70	0.68	−0.23	0.52	−0.44	−5.27	3.18	−1.66	0.08	0.07	1.08
L1 X Lexicality	−1.29	2.38	−0.54	0.60	0.45	1.35	7.07	3.77	1.88	−0.01	0.03	−0.34
Control predictors
Age of L2 acquisition^3^	−1.05	2.15	−0.49	0.39	0.43	0.91	1.69	1.82	0.93	−0.03	0.02	−1.42
Current L2 exposure^4^	−0.40	0.32	−1.26	0.00	0.06	−0.01	**−0.63**	**0.27**	**−2.34∗**	0.00	0.00	0.18
Testing order^5^	7.81	10.12	0.77	−2.53	2.01	−1.26	−15.45	8.57	−1.80	0.13	0.08	1.50
Intercept	15.69	22.88	0.69	−1.02	4.54	−0.22	11.38	19.41	0.59	0.09	0.19	0.47

	**LSS**			**FFL**			**FFD**			**FC**		
	**variance intercept**			**variance intercept**			**variance intercept**			**variance intercept**		
Random effects												
Participants		1003.29			39.52			699.80			0.07	
Items		4.70			7.56			136.10			0.20	
Residual		2272.05			80.16			5706.50			0.34	

*L1, first-language; L2, second-language; LMEM, linear mixed effect models.*^1^*Contrasts were treatment coded (German-L1 vs. German-L2); model assumes German-L2 as the baseline.*^2^*Contrasts were treatment coded (Words vs. Pseudo-Words); model assumes Words as the baseline.*^3^*Continuous (years).*^4^*Continuous (% time).*^5^*Contrasts were treatment coded (German to French vs. French to German testing orders); model assumes French to German order as the baseline.*^∗^*and in bold p < 0.05.*

**FIGURE 3:**
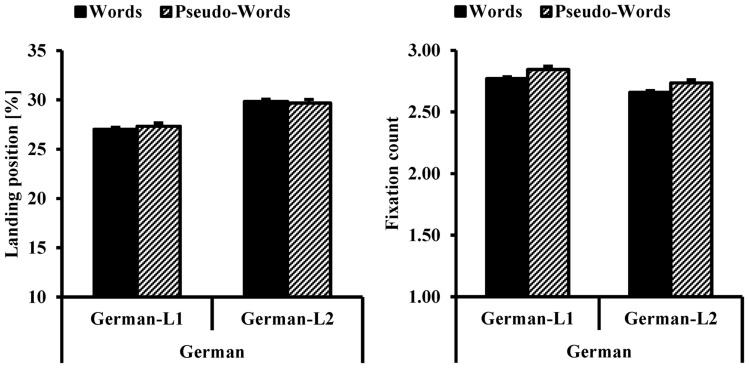
**Average Landing Position (%, on the left side) and total number of fixations (on the right side) as a function of bilinguals’ L1 (German-L1, German-L2) and lexicality (words, pseudo-words) in German**. The error bars represent the standard error of the mean.

**Figure 3** shows the average FFL and FC as a function of participants’ L1 and lexicality in German. Both graphs show no clear difference in results between participants’ L1s or types of lexicality.

### Reading Strategies in the Opaque Language (French) of Bilinguals

**Table 5** presents the LMEM output in French of LSS, FFL, FFD, and FC. In these sub-analyses, as expected, the LMEM showed a significant main effect of participants’ L1 in FFL [*F*(1,40.1) = 6.78, *p* = 0.013] and FC [*F*(1,40.4) = 11.47, *p* = 0.002], neither of which was yielded in the previous sub-analyses (this describes the two-way interactions evoked in the principal LMEM). The main effects indicate that French-L2 bilinguals fixated closer to the beginning of stimuli (26.34 vs. 32.28%) and made more fixations (2.85 vs. 2.53) than their French-L1 peers. In addition, there was a significant interaction between the participants’ L1 and lexicality on FC (β = –0.13, *SE* = 0.03, *t* = –4.31), showing an increased number of fixations for pseudo-words compared with words but only for the French-L1 bilinguals (β = 0.17, *SE* = 0.07, *t* = 2.48; 2.65 vs. 2.48). This interaction ends the description of the three-way interaction evoked in the principal LMEM. Moreover, as in the previous language analyses, a significant effect of the control predictor of current L2 exposure was observed for FFD [*F*(1,40.0) = 6.69, *p* = 0.013]; again, participants with less current exposure to their L2 presented longer FFD.

**Table 5:** French – βs, standard errors (SEs), and *t*-values for LMEM of the latency for sending the first fixation (LSS, millisecond), the first fixation location (FFL, %), the first fixation duration (FFD, millisecond), and the fixation count (FC) of stimuli.

	LSS			FFL			FFD			FC		
	β	*SE*	*T*	β	*SE*	*t*	β	*SE*	*t*	β	*SE*	*t*
Fixed effects												
L1^1^	−7.01	9.05	−0.77	**−5.81**	**2.29**	−2.53*	−1.75	9.65	−0.18	**0.35**	**0.09**	4.13*
Lexicality^2^	1.33	1.55	0.86	−0.27	0.53	−0.50	5.92	3.08	1.92	**0.17**	**0.07**	2.48*
L1 X Lexicality	−0.88	2.21	−0.40	−0.32	0.45	−0.70	−3.54	3.66	−0.97	**−0.13**	**0.03**	−4.31*
Control predictors												
Age of L2 acquisition^3^	−1.06	1.90	−0.56	0.54	0.48	1.12	0.69	2.02	0.34	−0.02	0.02	−1.34
Current L2 exposure^4^	−0.46	0.28	−1.63	0.00	0.07	0.04	**−0.77**	**0.30**	−2.59*	0.00	0.00	0.63
Testing order^5^	−1.44	8.96	−0.16	0.51	2.27	0.23	−3.95	9.53	−0.41	0.14	0.08	1.63
Intercept	21.95	20.26	1.08	−2.35	5.15	−0.46	9.77	21.56	0.45	−0.08	0.19	−0.43

	**LSS**			**FFL**			**FFD**			**FC**		
	**variance intercept**			**variance intercept**			**variance intercept**			**variance intercept**		
Random effects												
Participants		786.20			50.81			872.70			0.07	
Items		0.00			8.11			126.40			0.18	
Residual		1988.00			82.49			5440.20			0.35	

*L1, first-language; L2, second-language; LMEM, linear mixed effect models.*^1^*Contrasts were treatment coded (French-L1 vs. French-L2); model assumes French-L1 as the baseline.*^2^*Contrasts were treatment coded (Words vs. Pseudo-Words); model assumes Words as the baseline.*^3^*Continuous (years).*^4^*Continuous (% time).*^5^*Contrasts were treatment coded (German to French vs. French to German testing orders); model assumes French to German order as the baseline.*^∗^*and in bold p < 0.05.*

**FIGURE 4:**
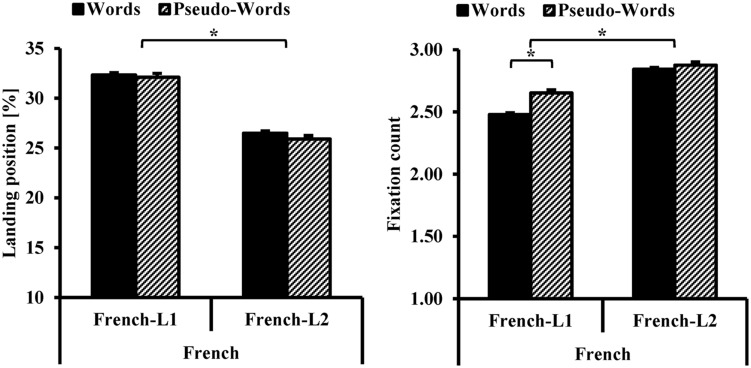
**Average Landing Position (%, on the left side) and total number of fixations (on the right side) as a function of bilinguals’ L1 (French-L1, French-L2) and lexicality (words, pseudo-words) in French**. The error bars represent the standard error of the mean. The ^∗^ represents a statistically significant difference with a *p* < 0.05.

**Figure 4** presents the average FFL and FC as a function of participants’ L1 and lexicality in French.

For FFL, **Figure 4** shows a difference between French-L1 versus French-L2 bilinguals. No difference is presented across lexicality; French-L2 bilinguals present an FFL close to the beginning of the words, while French-L1 bilinguals have an FFL close to the middle of the stimuli. The FC graph in **Figure 4** also presents a difference as a function of participants’ L1, with a higher number of fixations for French-L2 compared with French-L1 bilinguals and a higher number of fixations in pseudo-words than real words but in French-L1 bilinguals only.

## Discussion

The present study aimed to identify how language opacity and low proficiency level influence reading strategies in bilinguals. We explored reading strategies in two groups of bilinguals, one with a transparent (German) L1 and an opaque (French) L2, and the other with the opposite pattern. In both groups the readers’ proficiency in their L2 was low. Eye movement patterns were recorded while the participants were reading aloud words and pseudo-words presented in purely monolingual settings, so that they were deeply immersed either in a transparent (German) or an opaque (French) linguistic mode. The results revealed that the impact of language opacity was primarily observed on FFL and FC when bilinguals were reading in their L1. Whereas, the low proficiency level in their L2 yielded to the preferential selection of a specific reading strategy.

### Reading Strategies in the Transparent (German) and the Opaque (French) Languages of Bilinguals

The FC was higher and the FFL was closer to the beginning of stimuli in bilinguals who had a transparent rather than an opaque L1. This confirms our hypothesis regarding specific eye movement patterns in reading as a function of language opacity. This result, in line with previous research, suggests that transparent languages promote local and serial reading strategies, whereas opaque languages encourage more parallel and global strategies (Buetler et al., 2014, 2015). The FFL and the FC do indeed appear to determine whether the participants’ reading strategy consists of processing each letter linearly, as distinct graphemes to be converted into phonemes (local and serial strategy), or in reading words as portions of letters to be totally converted into phonemes (global and parallel strategy). The Dual Route Cascaded Model (Coltheart et al., 2001) proposes that reading using the lexical route involves the processing of all letters of words in parallel to find phonology. However, reading by means of the non-lexical route involves the assembling of letters serially (from left to right) into phonology. Our result, in line with the Dual Route Cascaded Model, shows FFLs closer to the beginning of words as well as an increased number of fixations in bilinguals with a transparent L1, and might suggest that they engage a serial process and therefore rely on the non-lexical route. In contrast, less FC and an FFL closer to the middle of the words would indicate that the global and parallel reading strategy, and thus the lexical route, is engaged when bilinguals with an opaque first language are reading. The lexical route engagement is also supported by previous results linking fixation in the middle of the word to lexical modulations (Vitu et al., 1990; Yao-N’dre et al., 2013).

### Reading Strategies in the Transparent Language (German) of Bilinguals

In this transparent language there were no statistical differences in bilinguals at any level. Finding no difference in the transparent language between bilinguals using a first dominant language and bilinguals using their second non-dominant language strongly supports and confirms our second hypothesis. The L2 of unbalanced bilinguals preferentially involves the non-lexical route, i.e., the use of a local and serial reading strategy for finding the pronunciation of words and pseudo-words. In line with the Dual Route Cascaded Model (Coltheart et al., 2001), finding no difference between the L1 and the L2 of bilinguals also supports our hypothesis that lexical-semantic knowledge is not relevant when reading in a transparent language. However, it is important to note that an impact of the transparent linguistic mode on L2 processing cannot be excluded. Indeed, in the present language its transparency is confounded with the low proficiency level of bilinguals.

### Reading Strategies in the Opaque Language (French) of Bilinguals

As expected, the FFL was closer to the middle of the words associated with a lower FC for French-L1 than for French-L2 bilinguals, confirming the association of local and serial reading strategies for their L2, and a more global and parallel processing by their L1. This difference between bilinguals when reading in their L1 versus their L2 supports the fact that weaker links between word forms and their lexical representation do indeed lead to the selection of a reading strategy based on the non-lexical route, as suggested by previous studies (Gollan et al., 2011; Fisher et al., 2012; Jeon, 2012). As such, the reading strategy in L2 is driven by the low proficiency level rather than by the opacity of the language. Interestingly, when reading words in this opaque language the FC was lower than when reading pseudo-words, but only in bilinguals with an opaque L1 (French-L1). When reading in French, French-L1 bilinguals were inclined to use a reading strategy strongly influenced by the linguistic mode (Levy et al., 2009; Jamal et al., 2012). This lexical effect supports the fact that the lexical route allows a global processing based on lexical-semantic knowledge, which plays a role in the process of finding the pronunciation of words. Notwithstanding, in de León Rodríguez et al. (2015), the lexical effect in the opaque language was observed in the FFL, which was not the case in the present study. One possible explanation for this difference may lie in the types of populations tested in each of these studies. In the present study, the population comprised bilinguals with a non-dominant L2 in whom the use of both reading strategies and reading routes was extremely unequal. In contrast, in de León Rodríguez et al. (2015) the group concerned bilinguals with equal proficiency level between their languages and, due to years of intensive practice, they were using both reading strategies at the same level. Consequently, the lexical processing of the stimuli is suggested to happen earlier in de León Rodríguez et al. (2015) work than in the present study.

Finally, in the main LMEM, the FFD presented a significant three-way interaction that was disseminated in marginal effects in each sub-model. This most likely implies a lexical marginal effect in French and the participants’ L1 by lexical marginal interaction in German. Worth noting, in linguistic stimuli the increase of the FFD has been previously associated with the increase of readers’ cognitive load (Hand et al., 2012; Radach and Kennedy, 2013). Therefore, in French, bilinguals tended to process words faster than pseudo-words (lower FFD). This may be interpreted as a result of a strong use of the lexical route due to the opacity of the language. In German, however, the marginal interaction showed a tendency toward faster processing of pseudo-words than words, but only for French-L1 bilinguals. This suggests that they were probably favoring the non-lexical route, not only because of the impact of the transparent language but also because of the dominant local and serial reading strategy of the non-dominant L2. In addition, there was also a main effect of current L2 exposure on FFD across all analyses (main- and sub-models), which does not change our main conclusions. This factor was included in order to assure that our results were not due to bias as proposed by previous bilingual studies in the field of eye movements in reading. Thus, increasing evidence indicates that current L2 exposure is closely linked to the reader’s proficiency level in a language (Whitford and Titone, 2012, 2015). Correspondingly, this result could be interpreted as if reading in a language to which the reader is less exposed would produce a greater cognitive load, and therefore longer FFD.

It is of high relevance to state that the parafoveal processing in the present study was possible for several reasons. Firstly, we presented single words with an eccentricity of 10° of the fixation point. Therefore, a direct comparison of our results with the literature on reading texts or sentences is somewhat limited. However, we have arguments that are in favor of our interpretation. We agree that the first fixation landing position in a word is mainly, yet not exclusively, driven by low-level visual features as word length (Rayner, 2009). The study of Fine and Rubin (1999a), for example, suggests that the number of characters rather than the visual angle is a better estimator for landing position of the saccade. In our study, the stimuli were presented using Courier New 72 pt. in bold as a font letter which correspond to a 1.35 of visual angle per letter. Thus, the distance between the fixation cross and the beginning of the stimulus was of 7.7 letters and the distance between the fixation cross and the end of the stimuli was of 12.7 (five letter stimuli) and 15.7 (eight letter stimuli); allowing some stimulus pre-processing (Rayner and Bertera, 1979; Rayner et al., 1979; Fine and Rubin, 1999a,b,c). Secondly, there is evidence for a large perceptual span in reading (Rayner, 1986; Rayner et al., 1989; Haikio et al., 2009; Rayner et al., 2009) for younger participants suggesting that a pre-processing of stimuli such as we used, is possible. Indeed, large reading span has been related to young (Rayner et al., 2009) and skilled (Rayner, 1986; Haikio et al., 2009) readers, especially in people without reading difficulties (Rayner et al., 1989). All these characteristics were part of our inclusion criteria. Furthermore, the parafoveal processing has been studied in text and sentence reading which consists of a more elaborated reading context (at least as far as the number of words and syntax in texts and sentences are concerned in the processing) than the single word reading aloud task we used. Finally, differences in landing positions between languages cannot be explained by a difference in complexity between both conditions. In fact this point was addressed in a prior reading study in balanced bilinguals, where there was no difference either in correct answers or in reaction times between languages (Buetler et al., 2014).

## Conclusion

Research on the modulation of reading strategies by language opacity (i.e., the complexity of the GPC rules) has attracted particular attention. Nevertheless, the existing data are still limited, especially in eye movement paradigms. The present study supports and confirms the hypothesis that different reading strategies, indexed by eye movement patterns, are associated with languages that have different degrees of opacity. When bilinguals read in their L1, if it is transparent, then they use a local and serial reading strategy relying on the non-lexical route. In contrast, if the L1 is opaque, then the bilinguals’ reading strategy is parallel and relies more on the lexical route. Moreover, the non-dominant L2 of bilinguals is being treated preferentially by a local and serial reading strategy, supporting the hypothesis that the reader’s proficiency also plays an important role in the reading strategy selection.

## Author Contributions

RM, J-MA, ML, DL, KB, designed research; DL, KB, NE performed research; DL, ML analyzed data; and RM, J-MA, ML, DL, KB, NE wrote the paper.

## Conflict of Interest Statement

The authors declare that the research was conducted in the absence of any commercial or financial relationships that could be construed as a potential conflict of interest.

## Supplementary Material

TABLE S1 **Characteristics of the stimuli**. The Table S1 presents the psycholinguistic factors (summated bigram frequency, neighborhood size and word frequency expressed in a log-transformed lexical frequency) for words and pseudo-words as a function of language (French and German) and length (five-letter and eight-letter).
